# Pseudoangiomatous Stromal Hyperplasia: A Rare Case of Bilateral Axillary Ectopic Breast Tissue

**DOI:** 10.7759/cureus.73450

**Published:** 2024-11-11

**Authors:** Natasha Natoli, Nicholas De Leo, Emanuella M Brito, Martin I Newman

**Affiliations:** 1 Surgery Department, Ross University School of Medicine, Bridgetown, BRB; 2 Plastic Surgery, Cleveland Clinic Florida, Weston, USA; 3 Surgery Department, Nova Southeastern University Dr. Kiran C. Patel College of Osteopathic Medicine, Fort Lauderdale, USA; 4 Plastic and Reconstructive Surgery, Cleveland Clinic Florida, Weston, USA

**Keywords:** axilla, breast, breast neoplasm, pash, pseudoangiomatous stromal hyperplasia

## Abstract

Pseudoangiomatous stromal hyperplasia (PASH) is a benign but rare mesenchymal proliferation of the mammary stroma, characterized by pseudovascular spaces within a hyperplastic matrix. PASH can be classified as either non-tumor-forming or tumor-forming. The non-tumor-forming type is an infiltrative and clinically undetectable mass, incidentally found in approximately a quarter of breast biopsies. Tumor-forming PASH, however, is a rare neoplasm of the breast parenchyma, with its occurrence in extra-mammary sites, such as the axilla, reported in less than a dozen cases.

We report the case of a 38-year-old female who presented with bilateral, progressively enlarging axillary growths. Following mammography, which classified the masses as breast imaging-reporting and data system (BI-RADS) 2, the patient underwent bilateral subcutaneous mastectomy for the excision of the axillary ectopic breast tissue. Final pathology revealed PASH with complete excision. This report highlights the importance of including PASH as a differential diagnosis for any mass presenting along the mammary ridge. Diagnosing and properly managing this rare neoplasm require a nuanced examination integrating clinical, radiological, and histopathological perspectives.

## Introduction

Pseudoangiomatous stromal hyperplasia (PASH) is a benign yet diagnostically challenging proliferative lesion of mammary stroma characterized by slit-like pseudovascular spaces within a hyperplastic stromal matrix. Of note, the pseudovascular spaces refer to the concern for true vascular spaces in the context of low-grade angiosarcoma [1]. There are generally two types of PASH: non-tumor-forming and tumor-forming [2]. Non-tumor-forming PASH is typically not considered a differential diagnosis for an axillary mass, as it is commonly an infiltrative and clinically undetectable incidental finding in the pathological examination of breast biopsy. Tumoral PASH, however, is uncommonly encountered in breast parenchyma, and its occurrence in extramammary sites, especially the axilla, remains an exceptionally rare phenomenon with fewer than a dozen clinical cases reported so far [3]. It usually presents as a mass or tumor and must be differentiated from other causes such as lymphadenopathy or a malignant tumor [4].

In this report, we present a case of axillary tumoral PASH found incidentally in the setting of progressively enlarging axillary growths for which the patient underwent elective excision. We also speculate on and entertain theories about the role of hormones in tumor formation, providing insights as to why premenopausal and postmenopausal women receiving hormone therapy are more predisposed to PASH. Diagnosis and proper treatment of this condition call for a nuanced examination integrating clinical, radiological, and histopathological perspectives [5].

## Case presentation

**Figure 1:**
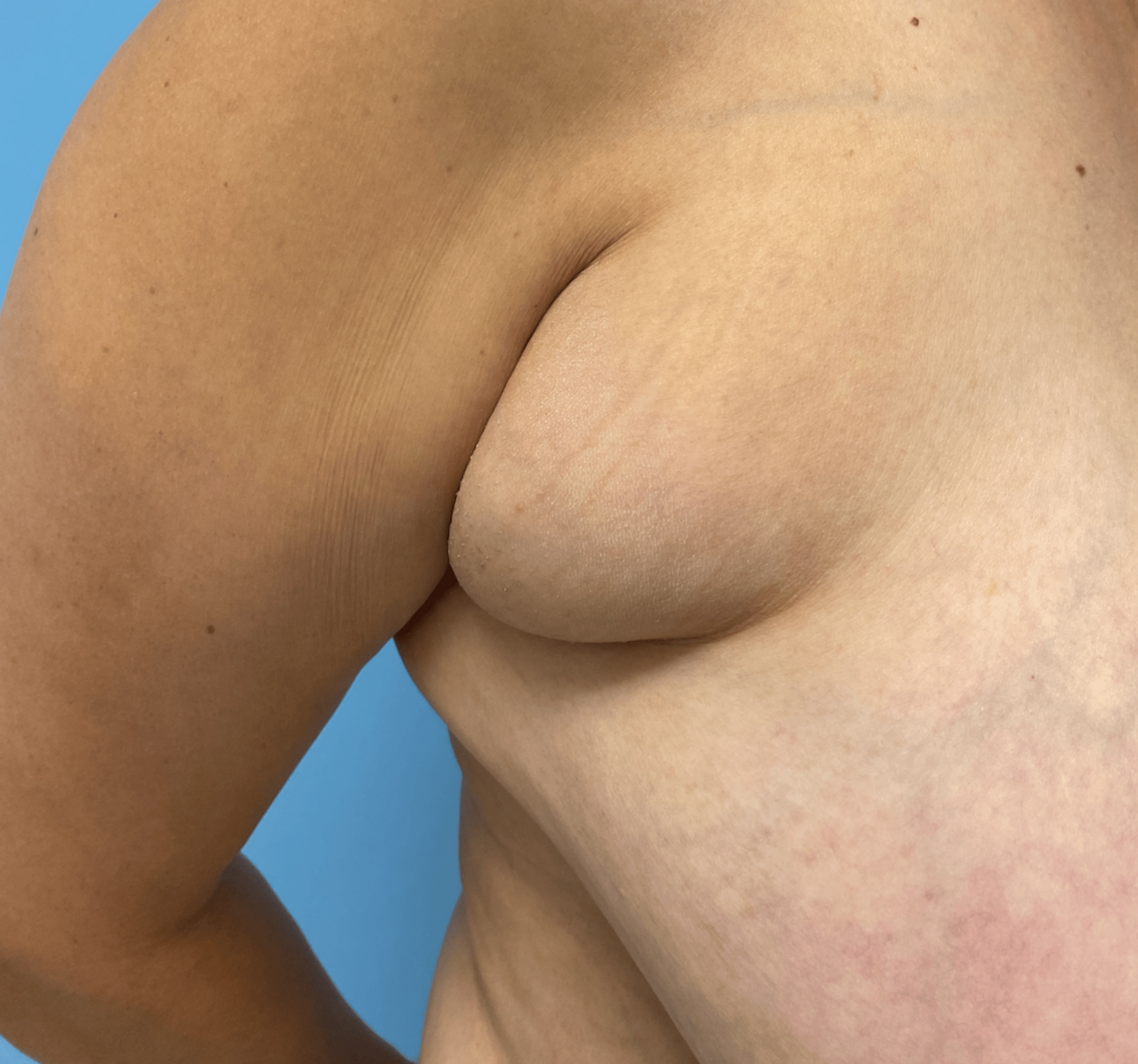
Clinical presentation of axillary pash Axillary PASH characterized by a well-circumscribed, firm mass in the axillary region PASH: pseudoangiomatous stromal hyperplasia

A 38-year-old premenopausal female with no past medical history presented to the clinic with a chief complaint of bilateral and progressively enlarging axillary growths (Figure 1). The patient noted that the masses were more tender during her menstrual cycle. She had a past surgical history of bilateral breast augmentation with subpectoral saline implants in 2002 and a cesarean section in 1998. The patient denied any history of breastfeeding and she was not taking any form of hormonal contraceptive. The patient was a lifelong nonsmoker. While she herself had no personal history of breast cancer, her family history was significant for breast cancer diagnosed in her sister at the age of 40 and a paternal aunt at the age of 60.

Physical Examination at initial presentation revealed asymmetry of the breasts, with grade 2 ptosis and grade 4 contracture of saline implants bilaterally. The right breast was tuberous, with a palpable, well-defined, rubbery 5-cm mass, noted at the anterior axillary line within the axilla. Examination of the left breast revealed a palpable, nontender, 4 cm soft, rubbery mass that was less defined along the left anterior axillary line. Palpable nodularity was also noted within the soft tissue of the left breast at the 12-1 o’clock position.

**Figure 2:**
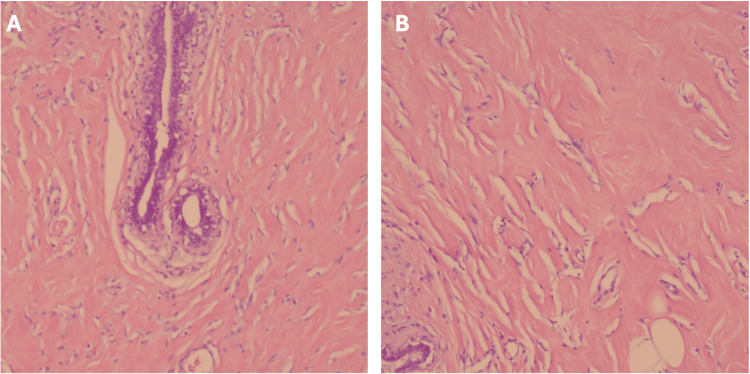
Histological examination of resected axillary growth Histological slides (A and B) of resected bilateral axillary mass confirming pathology of PASH with myofibroblast proliferation forming clefts that mimic vascular spaces PASH: pseudoangiomatous stromal hyperplasia

Subsequent preoperative diagnostic mammography revealed evidence of breast augmentation with intact bilateral subpectoral saline implants, scattered fibroglandular tissue, and additional axillary breast tissue bilaterally, more prominent on the right. There was no evidence of suspicious masses or lesions, skin thickening, or irregular architecture. These findings were classified as breast imaging-reporting and data system (BI-RADS) 2. The patient underwent bilateral subcutaneous mastectomy for excision of bilateral axillary ectopic breast tissue under general anesthesia. Pathology revealed PASH with complete excision (Figure 2). The final diagnosis was determined by combining imaging with excisional biopsy. Stains for CD34, CD31, and factor VIII were not conducted as the mass had benign characteristics both on imaging and pathology assessment. At a follow-up two weeks later, the patient showed no evidence of recurrence, infection, or wound breakdown.

## Discussion

First described in 1986, PASH is a common incidental finding on histologic examination of biopsied breast tissue, reported in nearly a quarter of specimens [4,6-8]. While the infiltrative form of PASH is well documented, the tumor-forming, nodular type of PASH is rare. Our review of the literature on cases of nodular PASH detected in aberrant breast tissue found within the axilla revealed less than a dozen cases [1,3,4,6-13].

**Figure 3:**
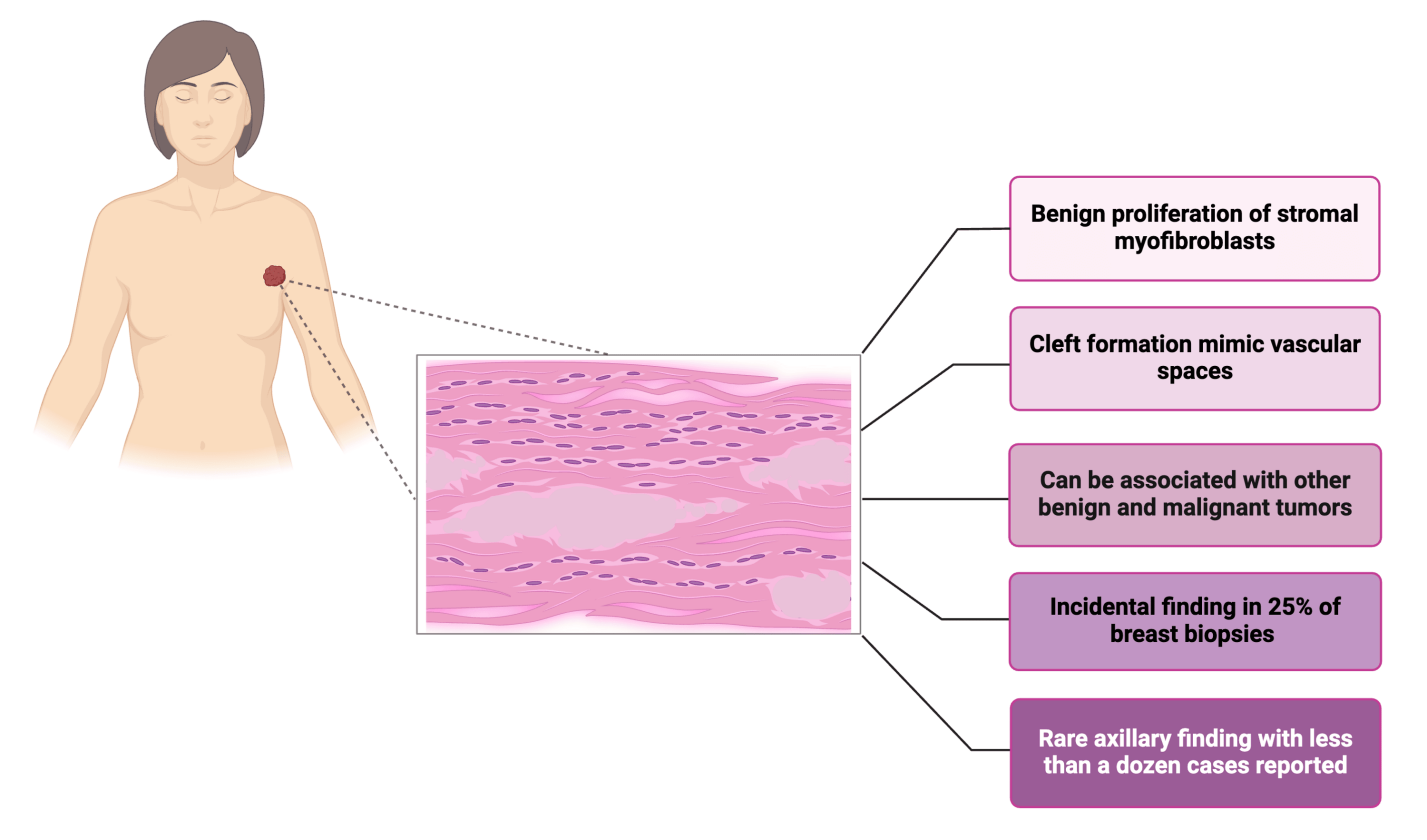
Histology and characteristics of axillary PASH Line diagram created in BioRender by Brito EM (www.BioRender.com/k02i904) PASH: pseudoangiomatous stromal hyperplasia

PASH is defined as a mesenchymal proliferative breast condition. The gross appearance of tumoral PASH resembles fibroadenoma, with a firm, rubbery, white fibrous surface [4]. On histological examination, one can appreciate the proliferation of spindle-shaped myofibroblasts, mimicking endothelial cells as they line slit-like spaces in the collagenous stroma. These spaces are devoid of red blood cells, as they are not true vascular channels. However, their resemblance to blood vessels warrants close examination and differentiation of PASH from low-grade angiosarcoma [6,7,13] (Figure 3). Immunochemical staining may be used to identify with higher specificity the origin of tissue. PASH stains positively for CD34, actin, vimentin, desmin, estrogen and progesterone receptors, and Bcl2 [10].

PASH is undiagnosable on imaging, as the tumoral form may resemble both benign and malignant growths. Due to its unclear clinical and radiological presentation, a core-needle biopsy is needed for diagnosis. Ultrasonography of tumoral PASH will demonstrate a well-circumscribed, hypoechoic, oval mass with mild vascularity. Mammography will illustrate a well-circumscribed, non-calcified, homogenous, dense mass [10]. MRI will demonstrate a well-circumscribed mass that exhibits a low signal in T1-weighted images and a distinctive heterogenous lace-like pattern in T2-weighted images [8,10]. Additionally, 4.1% of cases of tumoral PASH demonstrate cystic changes on imaging [4].

A comprehensive understanding of the embryological origins of the breast is crucial for exploring potential developmental aberrations that may lead to the formation of ectopic breast tissue. During the first trimester, symmetrical ectodermal thickenings extend bilaterally from the axilla to the groin, forming the mammary ridges [14]. These milk lines selectively recede, eliminating tissue outside the pectoral region to promote mammary gland development. Anomalies in this process of selective regression give rise to aberrant breast tissue [14]. This occurrence is found more often in women (2-6%) than in men (1%) and is bilateral in 30.3% of cases [12,13]. PASH has been reported in 24% of cases of gynecomastia in men [12].

By examining distinct features of this case while simultaneously comparing it to 10 other reports of PASH presenting in the axilla, we seek to enhance the current literature on PASH, shedding light on its atypical presentation in the axillary region and its association with aberrant breast tissue. A thorough review of the current literature showed less than a dozen cases of tumoral PASH within the axilla (Table 1). Of those cases, two were bilateral [1,13] and eight were unilateral [3,4,6-10,12]. Of the bilateral cases, one was reported as having synchronous growth [1] while the other was reported to have an asynchronous growth pattern [13]. As for the distribution of this pathology, it is predominantly found in premenopausal women and postmenopausal women taking hormone therapy. Of the 11 cases reported, eight were female [1,3,4,6-10,14] and two were male [12,13] with a ratio of 4:1. Among the female patients, five were premenopausal [1,6,7,9,10] and three were postmenopausal [3,4,8]. Of the postmenopausal patients, one was reported to be receiving hormone therapy for in vitro fertilization (IVF) [8]. Thus, it could be theorized that estrogen and progesterone play a unique but undiscovered role in the pathogenesis of tumoral PASH [15,16].

**Table 1:** Summary of previously reported cases of PASH PASH: pseudoangiomatous stromal hyperplasia

Study	Year	Patient age in years/gender	Clinicopathological Features	Management Strategies	Recurrence
Lee et al. [6]	2005	43/female	Unilateral axillary mass	Surgical excision	No recurrence at 1-year follow-up
Jordan et al. [4]	2011	52/female	Two unilateral axillary masses	Surgical excision	Recurrence status not reported
Vega et al. [13]	2014	44/male	Single bilateral axillary masses	Surgical excision	No recurrence at 1-year follow-up
Shimpi et al. [8]	2015	55/female	Unilateral axillary mass	Surgical excision	Contralateral breast invasive ductal cell carcinoma at 1-year follow-up
Alikhassi et al. [1]	2016	45/female	Bilateral axillary small nodules	Observation. Patient refused skin-sparing mastectomy	Follow-up status not reported
Canu et al. [9]	2018	20/female	Unilateral axillary mass	Surgical excision	No recurrence at 6-month follow-up
Val-Bernal et al. [12]	2018	38/male	Unilateral axillary mass	Surgical excision	No recurrence at 3-month follow-up
Liu et al. [3]	2019	52/female	Unilateral axillary mass	No surgical resection, interruption of estradiol medication	Recurrence status not reported
Maya et al. [7]	2022	42/female	Unilateral axillary mass	Surgical excision	No recurrence at 2-year follow-up

Current recommendations for biopsy-confirmed nodular PASH include follow-up imaging and consideration of surgical excision depending on size, associated symptoms, and growth rate. Up to 26% of nodular PASH cases within the breast parenchyma experience recurrence following surgery [9]. Of the 11 reported cases, 10 patients were managed with surgical excision [1,3,4,6-12]. One out of those 10 patients experienced a unilateral, ipsilateral recurrence five months post-excision [13]. One patient was managed non-surgically as she was asymptomatic and actively pursuing IVF. In this case, the patient discontinued estradiol, which resulted in a subsequent reduction in tumor size; however, she remained on progesterone for her IVF treatment [4]. Understanding the pathophysiology and presentation of tumoral PASH could aid clinicians in the diagnosis and proper management of this rare neoplasm.

## Conclusions

We discussed a rare case of a 38-year-old premenopausal female with bilaterally enlarging and tender axillary growths, highlighting the importance of including PASH as a differential diagnosis in patients presenting with an axillary mass. When considering the embryological origin of the breast, PASH may be included as a differential diagnosis for any mass presenting along the mammary ridge. However, beyond the differentials, a definitive diagnosis of PASH requires a biopsy or excision. In cases of tumoral PASH, surgical excision remains the gold standard for both diagnosis and curative treatment.
